# Behavioral and neurophysiological signatures of interoceptive enhancements following vagus nerve stimulation

**DOI:** 10.1002/hbm.25288

**Published:** 2020-12-16

**Authors:** Fabian Richter, Adolfo M. García, Nicolas Rodriguez Arriagada, Adrian Yoris, Agustina Birba, David Huepe, Heinz Zimmer, Agustín Ibáñez, Lucas Sedeño

**Affiliations:** ^1^ Department of Psychology University of Cologne Cologne Germany; ^2^ Universidad de San Andrés Buenos Aires Argentina; ^3^ National Scientific and Technical Research Council (CONICET) Buenos Aires Argentina; ^4^ Faculty of Education National University of Cuyo (UNCuyo) Mendoza Argentina; ^5^ Global Brain Health Institute University of California San Francisco California USA; ^6^ Faculty of Psychology University of Buenos Aires Buenos Aires Argentina; ^7^ Institute of Translational and Cognitive Neuroscience (INECO Foundation Favaloro‐University‐CONICET) Buenos Aires Argentina; ^8^ Center for Social and Cognitive Neuroscience, School of Psychology Universidad Adolfo Ibáñez Santiago Chile; ^9^ Universidad Autónoma del Caribe Barranquilla Colombia

**Keywords:** heartbeat detection task, heartbeat‐evoked potential, interoception, neurovisceral communication, noninvasive vagus nerve stimulation

## Abstract

An accruing body of research has shown that interoception (the sensing of signals from the body's internal milieu) relies on both a direct route (afforded by the vagus nerve) and a secondary route (supported by somatosensory mechanisms). However, no study has causally tested the differential role of these pathways, let alone via direct stimulation. To bridge this gap, we tested whether multidimensional signatures of interoception are modulated by noninvasive vagus nerve stimulation (nVNS). Sixty‐three participants were divided into an nVNS and a sham‐stimulation group. Before and after stimulation, both groups performed a validated heartbeat detection (HBD) task including a genuinely interoceptive condition (monitoring one's own heartbeat) and a control exteroceptive condition (tracking an aurally presented heartbeat). Electroencephalographic signals were obtained during both conditions to examine modulations of the heartbeat‐evoked potential (HEP). Moreover, before and after stimulation, participants were asked to complete a somatosensory heartbeat localization task. Results from the interoceptive condition revealed that, after treatment, only the nVNS group exhibited improved performance and greater HEP modulations. No behavioral differences were found for the exteroceptive control condition, which was nonetheless associated with significant HEP modulations. Finally, no between‐group differences were observed regarding the localization of the heartbeat sensations or relevant cardiodynamic variables (heart rate and or heart rate variability). Taken together, these results constitute unprecedented evidence that the vagus nerve plays a direct role in neurovisceral integration during interoception. This finding can constrain mechanistic models of the domain while informing a promising transdiagnostic agenda for interoceptive impairments across neuropsychiatric conditions.

## Graphical abstract

We tested cardiac interoception after noninvasive vagal nerve stimulation (nVNS). Cervical nVNS increased interoceptive accuracy and underlying neural modulations. Instead, cervical nVNS had no impact on somatosensory heartbeat perception. This supports the key role of the vagal pathway for neurovisceral integration.

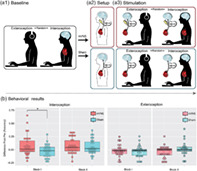

## 1 INTRODUCTION

Research on interoception, the sensing of signals from the body's internal milieu, is fundamental to constrain models of neurovisceral integrations (Craig, 2002a, 2002b). According to recent approaches (Couto et al., 2013; Craig, 2002a, 2002b; Critchley, Wiens, Rotshtein, Öhman, & Dolan, 2004; Khalsa, Rudrauf, Feinstein, & Tranel, 2009; Khalsa, Rudrauf, Sandesara, Olshansky, & Tranel, 2009), relevant processes hinge on two distinct pathways that may operate independently or interactively: one consists of visceral afferents projecting to the insular cortex (IC) (Craig, 2002a, 2002b; Pollatos, Schandry, Auer, & Kaufmann, 2007) and the anterior cingulate cortex (ACC) (Critchley et al., 2004), while the other involves skin afferents projecting to somatosensory cortices (Couto et al., 2013; Critchley, Mathias, & Dolan, 2001; Khalsa, Rudrauf, Feinstein, & Tranel, 2009; Khalsa, Rudrauf, Sandesara, et al., 2009). However, evidence on the function of these pathways is inconclusive. More importantly, no report has examined whether active stimulation of these pathways affects neurocognitive markers of interoception, leaving a major gap toward the formulation of mechanistic models. To address the issue, we assessed behavioral and electrophysiological signatures of interoception following direct electrical stimulation of a core neurovisceral route: the cervical vagus nerve.

Interoception can be examined in a qualified sense through studies on heart‐brain communication (Cameron, 2001; Craig, 2002a, 2002b; Critchley et al., 2004; Saper, 2002). Visceral afferents underlying cardiac awareness comprise vagal fibers which project to the IC—implicated in interoception (Craig, 2002a, 2002b) and other forms of body perception (Kirsch et al., 2020)—and the ACC—also implicated in interoceptive and body processing dynamics (Craig, 2002a, 2002b; Longhurst & Fu, 2012; Malliani, Lombardi, & Pagani, 1986). This is complemented by information from skin afferents projecting to somatosensory cortices (Cameron & Minoshima, 2002; Critchley et al., 2004; Khalsa, Rudrauf, Feinstein, & Tranel, 2009; Khalsa, Rudrauf, Sandesara, et al., 2009; Pollatos et al., 2007). So far, experimental findings on these pathways are restricted to two case studies focused on neurological and cardiological pathologies (Couto et al., 2013; Khalsa, Rudrauf, Feinstein, & Tranel, 2009; Khalsa, Rudrauf, Sandesara, et al., 2009). Yet, this evidence is undermined by its limited generalizability and the lack of direct manipulations of the visceral route to test its influence on pertinent neurocognitive markers. Promisingly, these limitations can be circumvented through noninvasive vagus nerve stimulation (nVNS).

Computational modeling and electrophysiological studies have shown that cervical nVNS can significantly activate vagal A‐ and B‐fibers that project to the nucleus tractus solitarius (NTS) and the parabrachial nucleus—that is, the main homeostatic integration sites in the brain stem (Mourdoukoutas, Truong, Adair, Simon, & Bikson, 2018; Nonis, D'Ostilio, Schoenen, & Magis, 2017). More particularly, imaging research on the neuromodulatory effects of cervical nVNS has revealed activation of classical primary vagal projection sites, including the IC, the NTS, the parabrachial area, the lateral primary somatosensory cortex (S1), the thalamus, and the caudate (Frangos & Komisaruk, 2017). These findings strongly suggest that cervical nVNS can activate vagal afferents and, at a cortical level, may modulate activity along interoceptive key areas. Indeed, incipient data (Villani, Tsakiris, & Azevedo, 2019) suggests that auricular nVNS can trigger interoceptive effects. However, such results may be undermined by the use of suboptimal interoceptive paradigms (such as the heartbeat counting and discrimination task), which have been criticized because of their working memory load, the potential for participants to over‐estimate their performance, and the possible role of attentional interference (Ehlers, Margraf, Roth, Taylor, & Birbaumer, 1988; Jones, 1994; Katkin, 1985; Katkin, Morell, Goldband, Bernstein, & Wise, 1982; Montgomery & Jones, 1984; Pennebaker, 1982; Phillips, Jones, Rieger, & Snell, 1999; Richards, Edgar, & Gibbon, 1996; Ring & Brener, 1996; Ring, Brener, Knapp, & Mailloux, 2015). The heartbeat detection (HBD) paradigm used by our team circumvents these shortcomings given that it is able to identify correct responses and does not require participants to keep track of their counting. On the other hand, the study by Villani et al. (2019) did not include any neurocognitive marker of cardiac interoception (such as the heart‐evoked potential [HEP]), nor an evaluation of the participants' somatosensory sensations.

Here, bridging these gaps, we manipulated the vagal activity of healthy individuals using cervical nVNS (or cervical sham stimulation) and examined its effects on neurophysiological and behavioral measures of interoception. Behavioral performance was assessed through a modified version of Schandry's HBD task, whereby participants track their heartbeats through key presses (Lerman et al., 2016; Silberstein et al., 2016). Cortical responses were investigated through analyses of the HEP, an event‐related potential (ERP) modulated by attention to cardiac signals (Schandry, Sparrer, & Weitkunat, 1986). Moreover, to determine whether nVNS affects somatosensory dynamics, we asked participants to locate their heartbeat sensations on a mannequin‐template before and after stimulation (Khalsa et al., 2018; Khalsa, Rudrauf, Feinstein, & Tranel, 2009; Khalsa, Rudrauf, Sandesara, et al., 2009).

Given that cervical nVNS activates cortical interoceptive areas, we expected a greater modulation of interoceptive markers (both behavioral and cortical) after this stimulation as compared to a cervical sham stimulation. Moreover, we predicted that the localization of felt heartbeat sensations between these different stimulations would not change since nVNS should not selectively influence any kind of somatosensory perception. In short, with this approach we aim to establish new experimental constraints for models of neurovisceral integration.

## 2 METHODS

### 2.1 Participants

The study comprised 76 participants, randomly assigned to the nVNS group or the sham‐stimulation group (sham group), as in previous research (Lerman et al., 2016; Silberstein et al., 2016). However, given the potential influence of demographical, physiological, cognitive, and mood variables on interoceptive sensitivity (Cameron, 2001; Jones, Jones, Rouse, Scott, & Caldwell, 1987; Mende‐Siedlecki, Said, & Todorov, 2013; Montgomery & Jones, 1984; Schandry & Montoya, 1996; Schandry & Specht, 1981), participants from both groups that did not match our inclusion/exclusion criteria (age between 18 and 45 years old, body mass index between 16 and 32, and minimum of 10 years of formal education) were excluded from the analyses upon completion of the datcollection process. In addition, we subsequently eliminated participants who did not meet the minimum requirement of one response during the interoceptive condition (baseline and/or poststimulation phase), or reported a history of substance abuse and/or current medication. The final sample was composed of 35 subjects (20 females) who received nVNS treatment and 28 (16 females) who received sham stimulation. The groups did not differ significantly in terms of gender, age, education, and body mass index (see Table 1 for complete demographics). Moreover, a standard clinical screening including psychological and neuropsychological assessments (Table 1) revealed that both groups were also matched in executive functioning and working memory (as assessed through the INECO Frontal Screening [IFS] battery (Torralva, Roca, Gleichgerrcht, López, & Manes, 2009)), as well as in mood (based on the Beck Depression Inventory II (BDI‐II) (Beck, Steer, Ball, & Ranieri, 1996)), anxiety levels (as tapped by the State‐Anxiety Scale of the State–Trait‐Anxiety Inventory [STAI] (Spielberger & Vagg, 1984)), and subjective experience of internal sensations (interoceptive sensibility) [measured with the Multidimensional Assessment of Interoceptive Awareness (Mehling et al., 2012)). None of the participants we included in the final sample reported a history of neurologic or psychiatric disorders, substance abuse, current medication, respiratory diseases, or heart diseases. All of the participants read and signed a written consent that stipulates the details of the study and allows for its publication, and they also gave written informed consent in accordance with the Declaration of Helsinki. The full protocol was approved by the ethics committee of the Institute of Cognitive Neurology—a host institution of the Institute of Cognitive and Translational Neuroscience.

**TABLE 1:** Demographic, neuropsychological, mood, and interoceptive sensibility measures

Variable	Group		Statistics
	nVNS	Sham	
Demographic results			
Gender (F:M)	20:15	16:12	*X* ^2^ = 0.00, *p* = 1.00*
Age	26.34 (5.38)	25.79 (5.63)	*F* = 0.16, *p* = .69, *ηp* ^2^ = .003^#^
Education	17.17 (2.27)	16.46 (2.05)	*F* = 1.65, *p* = .20, *ηp* ^2^ = .026^#^
Neuropsychological assessment			
IFS global score	25.26 (3.09)	25.85 (2.45)	*F* = 0.44, *p* = .51, *ηp* ^2^ = .011^#^
Mood and anxiety results			
BDI‐II	7.68 (5.57)	7.71 (5.98)	*F* = 0.00, *p* = .98, *ηp* ^2^ = .000^#^
STAI‐S	31.04 (5.24)	30.17 (4.75)	*F* = 0.37, *p* = .55, *ηp* ^2^ = .008^#^
Interoceptive control measures			
MAIA	87.44 (29.97)	95.74 (18.21)	*F* = 1.15, *p* = .29, *ηp* ^2^ = .025^#^
Body mass index	22.29 (3.02)	22.23 (2.98)	*F* = 0.01, *p* = 94, *ηp* ^2^ = .00^#^

Abbreviations: ANOVA, analysis of variance; BDI‐II: Beck Depression Inventory II; IFS: INECO Frontal Screening battery; MAIA: Multidimensional Assessment of Interoceptive Awareness; nVNS, noninvasive vagus nerve stimulation; STAI‐S: State Anxiety Index; Education: measured in years of formal education.

*Note*: Results are presented as mean (*SD*). The asterisk (*) indicates chi‐square analyses, whereas the pound sign (^#^) refers to one‐way ANOVAs.

### 2.2 General procedure and study design

**FIGURE 1:**
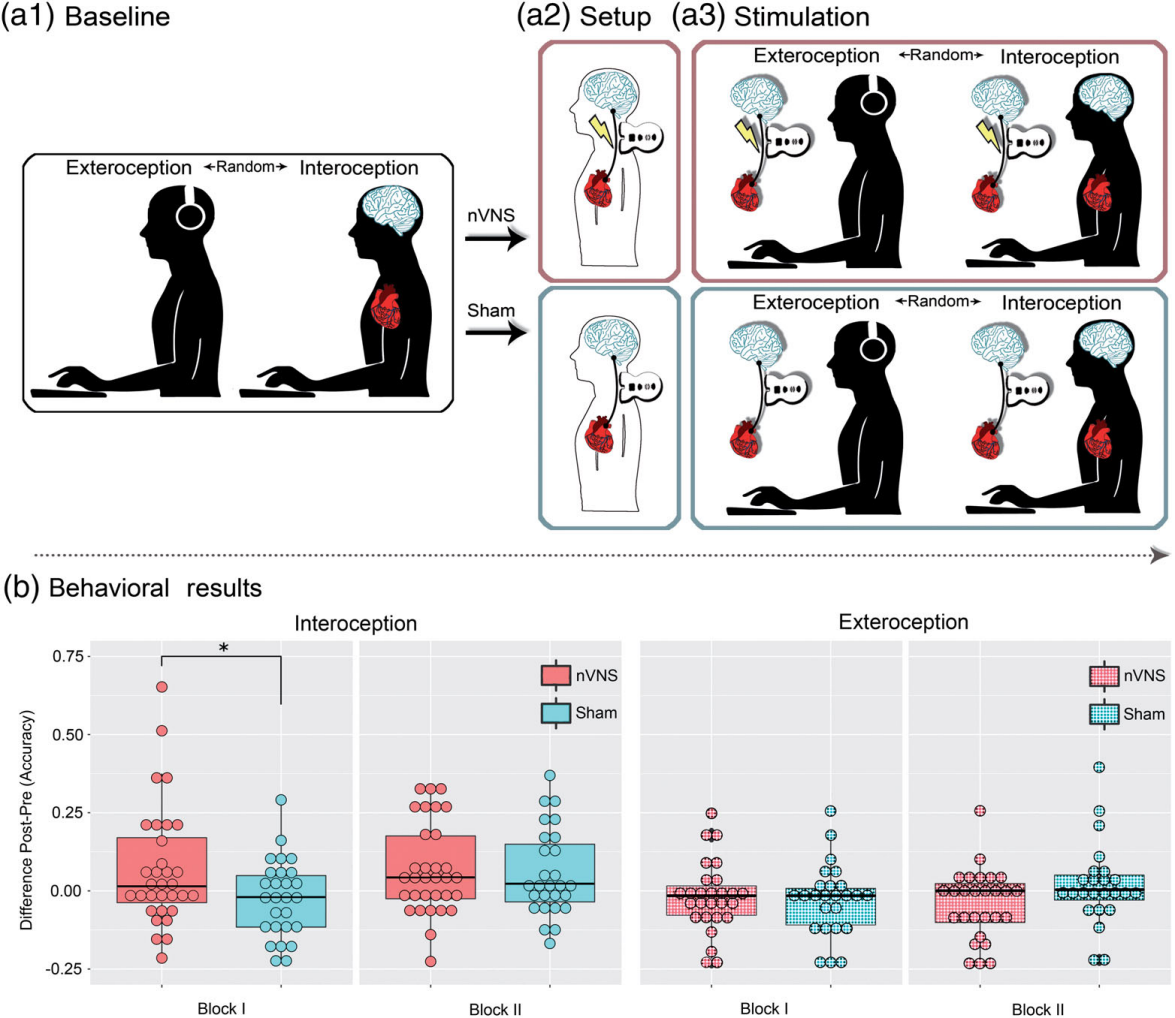
Study design and behavioral results on the heartbeat detection (HBD) task. (a) Study design. (a1) Baseline phase. Each participant, whether from the noninvasive vagus nerve stimulation (nVNS) or sham group, completed two consecutive exteroception blocks and two consecutive interoception blocks during the baseline phase. The order of the exteroception and interoceptive conditions was counterbalanced across participants. (a2) Setup. During the setup phase, the individual stimulation strength was determined separately for the right and left vagus nerve. The nVNS group received real stimulation of the vagus nerve, while the sham group received placebo stimulation. (a3). Stimulation phase. The stimulation phase was analogous to the baseline phase, but the vagus nerve in the nVNS group was bilaterally stimulated before each interoception or exteroceptive condition. The sham group received placebo stimulation before each condition. No stimulation took place during the HBD task. (b) Behavioral results (*N* = 63). The asterisk indicates significant differences (*p* < .05). The Y‐axis shows baseline and stimulation phase differences—that is, an individual subtraction value for each block (interoceptive or exteroceptive task) for each participant, indicating the change from pre‐to‐post stimulation condition, irrespective of the participant's baseline interoceptive accuracy. As expected, there was a significant effect of nVNS stimulation in the first but not the second interoceptive block

Participants were assessed in a sound‐insulating enclosure specially set up for electroencephalography (EEG) and electrocardiography (ECG) experiments. First, they underwent a clinical assessment (involving anxiety and depression scales, and the interoceptive sensibility questionnaire) and a neuropsychological evaluation through the IFS battery (details in Section 2.1). Then, EEG and ECG electrodes from a BioSemi Active‐two 128‐channel system were placed on each participant to evaluate interoceptive neurocognitive markers during the HBD task (details for the task in Sections 2.3.1 and 2.3.4). The first part of this task involves a baseline phase comprising an interoceptive and an exteroceptive condition (see Figure 1a1). Soon afterward, participants underwent a setup phase to determine the required strength of stimulation in each individual. The experimental group (nVNS group) received real cervical stimulation of the vagus nerve, while the control group (sham group) only feignedly received a cervical stimulation (details in Section 2.3.3 and Figure 1a2). Finally, participants received nVNS or sham stimulation (according to the level established in the setup phase) and performed the HBD task once again—note that stimulation was applied before each of the task's conditions: interoception and exteroception (see Figure 1a3). All subjects were blind to the experimental group (nVNS or sham). In fact, none of the participants knew at any time that there were two groups.

### 2.3 Study phases and HBD task data analysis

#### 2.3.1 Baseline phase

We used an adapted version of a validated HBD task (Canales‐Johnson et al., 2015; Couto et al., 2013; García‐Cordero et al., 2016; Melloni et al., 2013; Salamone et al., 2018; Sedeño et al., 2014; Yoris et al., 2015, 2018) in which participants were required to tap a key on a computer keyboard along with (a) their heartbeats (interoceptive condition) or (b) sequences of external stimuli (exteroceptive condition). In the interoceptive condition, subjects were asked to follow their own heartbeats in the absence of any sensory feedback for 2 min. This condition, which was performed twice in a row, provides an objective measure of each participant's interoceptive accuracy at baseline—that is, their objective performance in following their own heartbeats (Garfinkel, Seth, Barrett, Suzuki, & Critchley, 2015). In the exteroceptive condition, participants were instructed to follow an audio recording of a simulated heartbeat for 2 min. This was done also twice in a row. Note that the exteroceptive condition was strategically included to distinguish between strictly interoceptive modulations from a more general attentional effect (García‐Cordero et al., 2017; Petzschner et al., 2018).

During all blocks (interoceptive or exteroceptive task), participants were requested to respond with their dominant hand, to avoid excessive blinking or moving, and to keep their eyes on a fixation cross that remained visible on a screen throughout the whole experiment. They were instructed and checked to remove their wristwatches or bracelets and not to feel their pulse mechanically—for example, by sensing their wrist or carotid artery. The two conditions (interoception and exteroception) were counterbalanced across participants. During this phase, EEG and ECG signals were recorded with a BioSemi Active‐two 128‐channel system (see Sections 2.5 and 2.6).

#### 2.3.2 Setup phase

In the setup phase, subjects were shown a 2‐min video in which a person demonstrates correct usage of the nVNS device, but without giving information about its potential sensory effect or mode of action. Afterward, all participants received training regarding how to use the device (“It is a noninvasive, relatively simple, safe, painless, and well tolerated stimulation technique; a tingling sensation where the device is applied is normal, but should not cause major discomfort; these effects stop immediately once the stimulation session has been completed”) (Barbanti et al., 2015). Thereupon, participants placed the active device (nVNS or sham device) over the vagus nerve (at the neck over the arteria carotis communis; for localization, subjects palpated the pulse of their carotid artery) and increased the stimulation intensity by pressing a “plus button” until they felt a contraction in the area without pain or discomfort. The maximum intensity chosen by the participants was marked down by the experimenter (to preset the device with this intensity in the following stimulation phase) and the procedure was repeated for the contralateral side. As in other studies using nVNS, both sides were stimulated (Barbanti et al., 2015; Grazzi et al., 2016; Kinfe et al., 2015). Also, stimulating both sides seems desirable so as not to overlook possible (and yet explored) nVNS effects on interoception. To avoid possible laterality effects, the initial stimulation site (left or right vagus nerve) was counterbalanced across participants (for a detailed view of the counterbalanced schemes, also see Supplementary Tables 3 and 4).

#### 2.3.3 Stimulation phase

The stimulation phase started after a 5‐min resting period, as previous evidence suggests that the stimulation effect over the NTS washes out after this time interval (Frangos, Ellrich, & Komisaruk, 2015). The stimulation phase was identical to the baseline phase, with the exception that nVNS or sham stimulation took place once immediately before the interoceptive condition, and once immediately before the exteroceptive one. In both cases, the left and right vagus nerve were stimulated for 2 min each (Tassorelli et al., 2018) with the maximum intensity determined in the setup phase for each participant. Again, the initial side of stimulation (left or right) was counterbalanced across subjects. Also during this phase, we used the BioSemi Active‐two equipment to acquire the EEG and ECG signals (see Sections 2.5 and 2.6). We have chosen not to re‐stimulate between the two blocks of each condition, in order to be able to roughly estimate the duration of the predicted effect. If the effect peaks immediately after stimulation and then decreases again, as suggested by the results of Frangos et al. (2015), then any effect should be found mainly in the first block.

The nVNS device (GammaCore, provided by electroCore, LLC, Basking Ridge, NJ) is a handheld, portable appliance that employs a constant voltage‐driven signal consisting of a 1‐ms burst of 5‐kHz sine waves repeated at a frequency of 25 Hz (i.e., every 40 ms) (Nesbitt, Marin, Tompkins, Ruttledge, & Goadsby, 2015; Tassorelli et al., 2018). The device delivers a maximum voltage of 24 V and a maximum output current of 60 mA, whereby the stimulation amplitude is adjusted by the user. Stimulations are delivered transcutaneously in the region of the cervical branch of the vagus nerve through two stainless steel disc electrodes that are manually coated with a conductive gel. Stimulation of the neck in the region of the right and left cervical vagus nerve lasted 120 s for each side. In both treatment group (sham and nVNS), the examiner instructed the subject to place the device just under the angle of the mandible, lateral to the trachea, and medial to the sternocleidomastoid. The sham device is identical in appearance, weight, visual and audible feedback, and it was used for the same period of stimulation. Crucially, stimulation parameters of the sham device were set for a low‐frequency (0.1 Hz) biphasic signal with maximum range of ±14 V, which produces a slight tingling sensation on the skin but does not stimulate the vagus nerve or cause muscle contraction (Lerman et al., 2016; Silberstein et al., 2016).

#### 2.3.4 HBD task data analysis

Behavioral performance on the HBD task was analyzed for each subject through a modified version of Schandry's (Schandry, 1981) precision index (Couto et al., 2015; García‐Cordero et al., 2016; Melloni et al., 2013; Yoris et al., 2015, 2018). This index is based on correct responses and recorded heartbeats. The latter refers to the total number of heartbeats recorded for each block of each condition. Correct answers refer to the total number of responses that matched each of the subject's heartbeats. To estimate this match, every motor response is assessed for proximity relative to its preceding heartbeat; if the motor response falls within a given time window of any heartbeat, this response is considered correct. Here, the length of the time window is determined by the subjects' heart rate (HR). The exact procedure to estimate the time window for each subject is described in detail in earlier reports (García‐Cordero et al., 2016, 2017; Yoris et al., 2018) and in the Supplementary data. The behavioral precision or accuracy index was then calculated following this equation:

$$\frac{1-\left(\text{Recorded heartbeats}-\sum \text{Correct answers}\right)}{\text{Recorded heartbeats}}$$

This accuracy index can vary between 0 and 1, with higher scores indicating small differences between the total number of correct answers and recorded heartbeats, and, thus, better performance.

To analyze this accuracy index, first, an individual subtraction score was calculated for each block of each condition per subject by subtracting the baseline score from the stimulation score. This allowed us to ascertain the actual change of each participant after stimulation, irrespective of his or her initial interoceptive accuracy. To compare this subtraction score between the groups, we applied a robust permutation test (5,000 permutations, *p* < .05), which proves adequate as it is blind to the distribution of the observations under the null hypothesis (Nichols & Holmes, 2002). The data from each comparison between groups were separately subjected to a random partition and a *t*‐value was then calculated. This process was repeated 5,000 times to construct a *t*‐value distribution under the null hypothesis. The null hypothesis was rejected if a resulting *t*‐value was greater than the most extreme 5% of the distribution (*p* < .05, two tailed *t* test) (García‐Cordero et al., 2017). Given that this approach does not allow for a within‐factor design, and that our objective was to detect the block yielding a possible stimulation effect, each block was analyzed individually. Also, analyzing the blocks individually enabled us to provide an approximate estimate for the duration of the effect. Given the discrete nature of HBDT data, and as shown in previous studies (e.g., Canales‐Johnson et al., 2015; Couto et al., 2013; de la Fuente et al., 2019), one block (120 s) is enough to elicit robust behavioral data. Yet, both blocks were taken together for HEP analyses, because EEG data are prone to various artifacts that are magnified when the signal is not sufficiently long (Korats, le Cam, Ranta, & Hamid, 2013).

We also conducted an additional analysis to confirm that nVNS actually increased interoceptive accuracy relative to baseline and that the effect found in the first block of the interoceptive condition was not driven by a negative influence of sham stimulation on interoceptive sensitivity. To this end, we calculated the mean pre–post differences of the accuracy scores and conducted paired pre–post permutation analyses separately for both groups and both blocks. Also, to rule out an effect of baseline discrepancies between groups, we calculated the mean baseline differences between them, separately for both blocks. Effect sizes were determined using Cohen's *d* (Cohen, 1988).

### 2.4 Heartbeat perception localization

To analyze the locations of perceived heartbeat sensations, in line with previous procedures (Khalsa et al., 2018; Khalsa, Rudrauf, Feinstein, & Tranel, 2009; Khalsa, Rudrauf, Sandesara, et al., 2009), we asked participants to locate where they felt their heartbeat on a mannequin‐template. This was done after the baseline and the stimulation phase of the interoceptive condition. Two regions of interest (ROIs) were defined from the most pinpointed zones (see Figure 3) and corresponding masks were created. The first consisted of the head and neck area, and the other one comprised the chest area. Then, all marked areas (equal to one) inside each mask were summed for every subject for both baseline and stimulation phases separately. Subsequently, a heartbeat location score was calculated for each individual by subtracting stimulation and baseline phases for each mask. Two permutation tests (5,000 permutations, *p* < .05) were performed on baseline‐corrected data (subtracting the baseline from the stimulation condition) to compare the heartbeat localization score of the two groups in both ROIs.

### 2.5 Cardiac measures: HR and HR variability during the HBD task

We performed complementary analyses to control for a possible efferent effect of nVNS on the autonomic nervous system and, hence, on interoceptive performance. To this end, we compared the differences between baseline and stimulation measures of HR and HR variability (HRV) between both stimulation groups (sham and nVNS) via permutation analyses (see for details of this statistical analysis in Section 2.3.4). For the analysis of HRV, beat‐to‐beat RR intervals from the ECG (using the MATLAB platform (MATLAB 2017b, The MathWorks, Natick, MA)] were imported to the HRV Analysis Software (HRVAS, Ramshur, 2010), and the root mean square of the successive differences of the beat‐to‐beat RR intervals was calculated. This time‐based analysis showed reliability over short recordings (<120 s) (Thayer, Åhs, Fredrikson, Sollers, & Wager, 2012) and is considered to reflect vagal cardiac influence in the absence of respiratory interference (Laborde, Mosley, & Thayer, 2017; Thayer et al., 2012). The HRVAS also created mean HR measures.

HR and HRV outcomes were compared between the stimulation groups by first subtracting the baseline from the stimulation condition to obtain a difference score at the individual level (following the same procedure applied for the HBD task, Section 2.3.4.). Thereafter, group differences were examined through the Monte Carlo permutation analyses (5,000 permutations, *p* < .05).

### 2.6 ERP measures

#### 2.6.1 EEG data recording and preprocessing

During the HBD task, EEG and ECG signals were recorded with a BioSemi Active‐two 128‐channel system at 1,024 Hz. Data were resampled offline at 256 Hz. To remove undesired frequencies, data were also band‐pass filtered during recording (0.1–100 Hz) and resampling (0.5–50 Hz). The reference was set by default to linked mastoids, and rereferenced offline to the average of the electrodes. To record ECG data, two additional electrodes were included, one attached to the lower left abdominal quadrant and one under the right collarbone.

All subsequent ERP analyses were run over a subset of participants (nVNS group: *n* = 27, 17 female; sham group: *n* = 28, 16 female) for whom artifact‐free EEG datasets were obtained. Reasons for exclusion were excessive movement during EEG recording that led to “noisy recordings.” This did not affect the similarity of the two groups in terms of gender, age, education, BMI and neuropsychological, mood or anxiety measures; see Supplementary data for the corresponding version of Table 1 (Supplementary Table 1).

In the preprocessing stage, data with eye movement contamination and cardiac field artifacts (CFAs) were removed using independent component analysis (ICA) as well as a visual inspection procedure, and thus excluded from further analyses. The CFA is generated during the contraction of the heart muscle and can be measured on the entire body surface, including the scalp. It is mainly observed during the QRS complex and in an attenuated form during the T wave (Dirlich, Vogl, Plaschke, & Strian, 1997; Kern, Aertsen, Schulze‐Bonhage, & Ball, 2013) (Dirlich et al., 1997; Kern et al., 2013). Also, as done in previous studies (García‐Cordero et al., 2016; Yoris et al., 2017), “noisy epochs” were detected through a visual inspection procedure and removed from the analyses (see Supplementary Table 2 for the corresponding statistics of the remaining epochs). The individual blocks of each condition (interoception and exteroception, each separately) were merged to provide a more robust, consistent, and reliable database for subsequent analyses, as also done in previous studies (García‐Cordero et al., 2017; Salamone et al., 2018; Yoris et al., 2018).

#### 2.6.2 ERP data analysis: HEP

The HEP is a voltage modulation emerging ~200–500 ms after the R‐wave peak (Canales‐Johnson et al., 2015; Fukushima, Terasawa, & Umeda, 2011; Montoya, Schandry, & Müller, 1993; Pollatos & Schandry, 2004). HEPs were established by sampling EEG epochs time‐locked to the R‐wave of the ECG, whereby the R‐wave detection was achieved by the Peakfinder function implemented in MATLAB (Yoder, 2009). EEG data were segmented between −200 and 500 ms relative to the R‐wave, and the epochs were subsequently baseline‐corrected (baseline: −200 to 0 ms) (Szczepanski et al., 2014).

Point‐by‐point comparisons along the thus calculated HEP signal were made via the Monte Carlo permutation test (5,000 permutations, *p* < .05) combined with bootstrapping (Manly, 2007), as done in previous studies on the HEP (Couto et al., 2015; García‐Cordero et al., 2016; Yoris et al., 2017; Yoris et al., 2018) and other ERPs (Amoruso et al., 2014; Andrillon, Kouider, Agus, & Pressnitzer, 2015; Naccache et al., 2005). The permutation test applied offers a straightforward solution for multiple comparison problems and does not depend on multiple comparisons correction or Gaussian assumptions about the probability distribution of the data (Nichols & Holmes, 2003). In addition, it avoids the selection of narrow a‐priori windows for analysis, preventing circularity biases (García‐Cordero et al., 2017; Yoris et al., 2018). Specifically, we used this data‐driven approach to evaluate each point of the signal from 200 to 500 ms, covering the typically HEP latency (Canales‐Johnson et al., 2015; Montoya et al., 1993; Pollatos & Schandry, 2004). Moreover, despite the implementation of ICA to reduce the impact of CFAs, the HEP signal was analyzed only after the 200‐ms mark (see below) to avoid the influence of this artifact, as previous reports suggested a possible contamination of the signal by the CFA before such time‐point (Kern et al., 2013; Park, Correia, Ducorps, & Tallon‐Baudry, 2014). Another theoretically conceivable confound, the motor potential, was previously shown to have no effect on the HEP during a HBDT (Salamone et al., 2018). To assess whether modulations of this ERP differed within groups, HEP curves from the baseline and stimulation phases were contrasted separately for the nVNS and the sham group. In addition, between‐group comparisons were calculated by considering the pre–post subtraction results from each sample—that is, HEP modulation in the baseline phase was subtracted from the stimulation modulation in the nVNS and the sham samples (Yoris et al., 2017).

**FIGURE 2:**
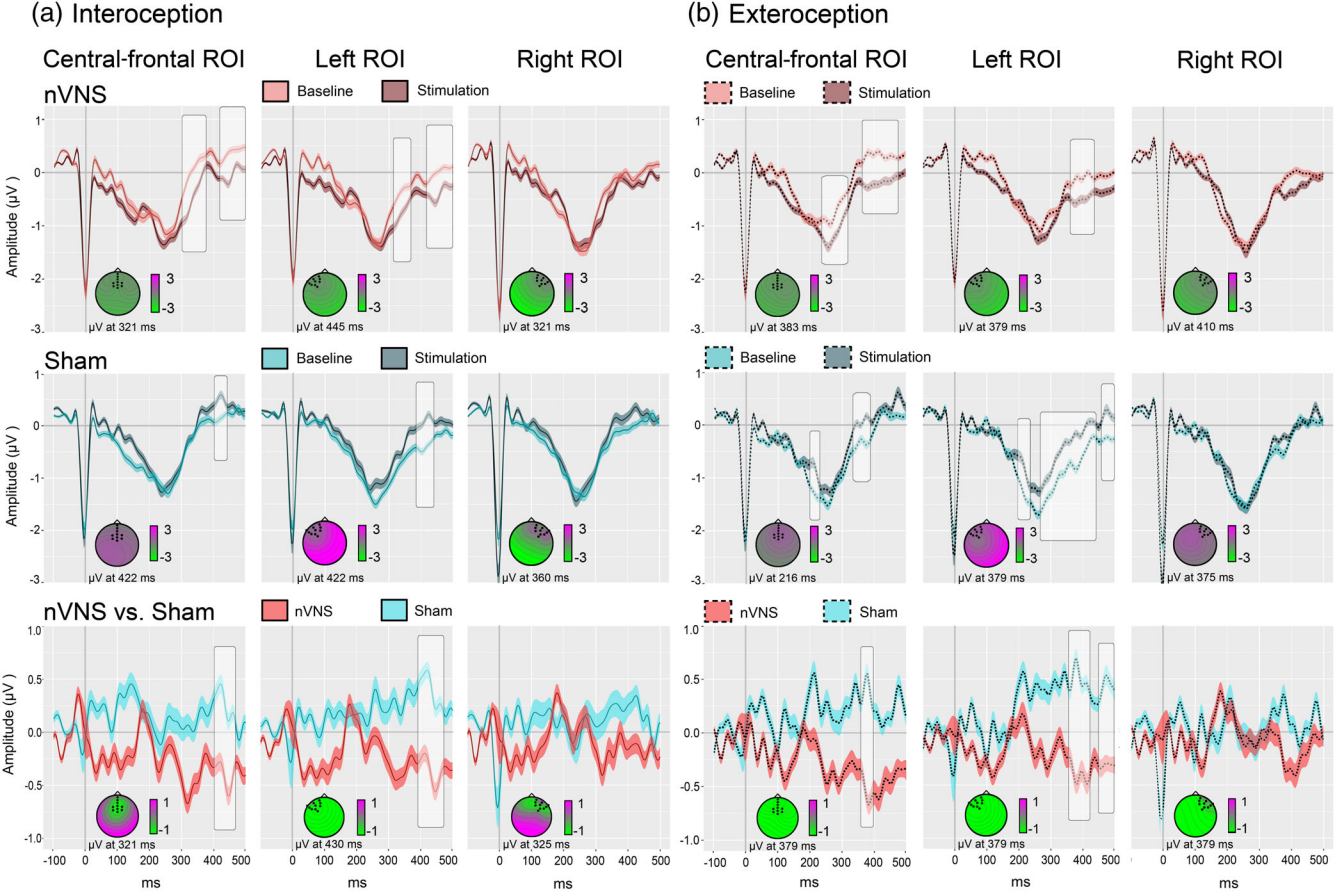
Heartbeat‐evoked potential (HEP) analysis. All differences reported were calculated via Monte Carlo permutations analyses (5,000 permutations, *p* < .05), performed point by point (Manly, 2007). A minimum extension of five consecutive points (=19.5 ms) was selected as criterion to identify clusters while avoiding noisy results (i.e., differences that failed to sustain in time). *N* = 55. Shadowed lines indicate *SD*. Brightened boxes indicate significant differences. ms, milliseconds, nVNS, noninvasive vagus nerve stimulation; ROI, region of interest

In light of the frontal topography of the HEP (Fukushima et al., 2011; Gray et al., 2007; Pollatos & Schandry, 2004), and following previous reports (García‐Cordero et al., 2016; Pollatos, Kirsch, & Schandry, 2005b; Pollatos & Schandry, 2004; Yoris et al., 2018), we selected three frontal ROIs: a right frontal ROI (BioSemi C3, C4, C5, C6, C7, C9, C10, C13, C14, C15); a left frontal ROI (BioSemi C26, C27, C28, C31, C32, D3, D4, D5, D6, D7); and a central‐frontal ROI (BioSemi C11, C12, C18, C19, C20, C21, C22, C23, C24, C25)—see Figure 2. To avoid noisy results a minimum extension of five consecutive points (i.e., 19.5 ms) which reached significance was selected as criteria to report clusters (García‐Cordero et al., 2017; Yoris et al., 2018).

### 2.7 Behavioral reaction times

Circular statistics were employed to analyze the temporal distribution of the key presses (Galvez‐Pol, McConnell, & Kilner, 2020; Kunzendorf et al., 2019; Ohl, Wohltat, Kliegl, Pollatos, & Engbert, 2016). For each motor response within an epoch, we calculated the temporal distance to the preceding R‐peak. Then, using Rayleigh tests (Landler, Ruxton, & Malkemper, 2018), we examined whether the temporal distribution of the average reaction times differed from a uniform distribution in each block of each condition. Also, we applied pairwise Watson tests to assess differences in response timing between baseline and stimulation phase for individual blocks and groups (Mardia & Jupp, 2008).

## 3 RESULTS

### 3.1 HBD task results

The permutation analysis of the HBD performance data (accuracy) revealed a significant between‐group difference in the subtraction score (stimulation phase minus baseline phase) for the first interoceptive block (*p* = .03, *d* = 0.52), but not for the second one (*p* = .88, *d* = .034). Concerning the exteroceptive control condition of the HBD task, no significant between‐group performance differences were observed in either block: Block 1: *p* = .55, *d* = 0.16; Block 2: *p* = .29, *d* = 0.29 (see Figure 1b).

All pre–post comparisons showed better performance after stimulation—with the exception of the first block of the sham group, which yielded nonsignificant results (Supplementary Table 6). There were no significant between‐group differences in the baseline levels for either the first or second block (see Supplementary Table 7).

### 3.2 HR and HRV results

For the interoceptive condition, no significant differences in HR were found between groups, neither in the first nor in the second block, when comparing the subtraction between the baseline and stimulation phases (Block 1: *p* = .34, *d* = .25; Block 2: *p* = .24, *d* = −.31). The same pattern was observed in the exteroceptive condition. Again, no significant difference between groups emerged (Block 1: *p* = .6, *d* = .14; Block 2: *p* = .4, *d* = .23). Furthermore, no significant associations were found between HR and HEP data when calculating Spearman correlations (see Supplementary Data, Table 8).

Regarding HRV during the interoceptive condition, no significant between‐group differences were found when comparing the baseline and stimulation subtractions for the first and the second block (Block 1: *p* = .75, *d* = .09; Block 2: *p* = .33, *d* = .27). For the exteroceptive condition again no significant difference was found in the first block (Block 1: *p* = .86, *d* = .06), however such a difference was found for the second block (Block 2: *p* = .02, *d* = .66). Here, again, no significant associations were observed between HRV and HEPs modulations when calculating Spearman correlations (see Supplementary Data, Table 9).

### 3.3 ERP results

During the interoceptive condition of the HBD task, in the nVNS group, HEP modulations at the central‐frontal and the left frontal ROI were significantly more negative *after* stimulation (central‐frontal ROI: 303–362 ms, *p* = .01; 432–499 ms, *p* = .01; left frontal ROI: 315–358 ms, *p* = .01; 432–499 ms, *p* = .01), whereas they were significantly more negative *before* stimulation in the sham group (central‐frontal ROI: 405–436 ms, *p* = .02; left frontal ROI: 354–370 ms, *p* = .03; 393–440 ms, *p* < .01) (see also Figure 2, Panel a).

A similar opposite pattern between the nVNS and the sham group was observed during the exteroceptive control condition of the HBD task. In the nVNS group, HEP modulations at the central‐frontal and the left frontal ROI were significantly more negative within the expected time window (200–500 ms) *after* stimulation (central‐frontal ROI: 249–264 ms, *p* = .03; 280–311 ms, *p* = .02; 370–432 ms, *p* = .01, and 448–463 ms, *p* = .03; left frontal ROI: 374–393 ms, *p* = .02; 409–424 ms, *p* = .02; 479–499 ms, *p* = .03). In the sham group, the opposite effect was observed. HEP modulations at the central‐frontal and the left frontal ROIs were significantly more negative *before* stimulation (central‐frontal ROI: 202–221 ms, *p* = .01; 339–354 ms, *p* = .02; 370–389 ms, *p* = .01; left frontal ROI: 202–229 ms, *p* = .01; 249–288 ms, *p* = .02; 303–448 ms, *p* = .01; 456–499 ms, *p* = .01) (see also Figure 2, Panel b).

In the group comparison, Monte Carlo permutations showed that, relative to the sham group, participants who received nVNS presented increased HEP differences between the stimulation and baseline phases during the interoceptive condition (central‐frontal: 409–463 ms, *p* = .04; left frontal ROI: 397–467 ms, *p* = .02). The same was true for this group during the exteroceptive condition (central‐frontal ROI: 370–393 ms, *p* = .01; left frontal ROI: 366–428 ms, *p* = .02; 456–499 ms, *p* = .02). For a summary of the averaged HEP values for the significant time windows, please see Supplementary Table 5. For Spearman's correlations between electrophysiological and behavioral data, replicating the findings of Pollatos and Schandry (2004), please see Supplementary Table 10.

In summary, the negative amplitude of the averaged HEP modulation in the nVNS group at the central‐frontal and the left frontal ROIs was significantly increased after stimulation. In the sham group, the opposite effect was seen in these ROIs: the negative amplitude of the HEP decreased after stimulation. Note that all significant modulations reported here lie within the canonical HEP time window (Kern et al., 2013). The baseline‐corrected comparisons between the two groups confirm significant differences between them. With regard to the right frontal ROI, no significant difference could be found in any of the comparisons.

### 3.4 Heartbeat localization

**FIGURE 3:**
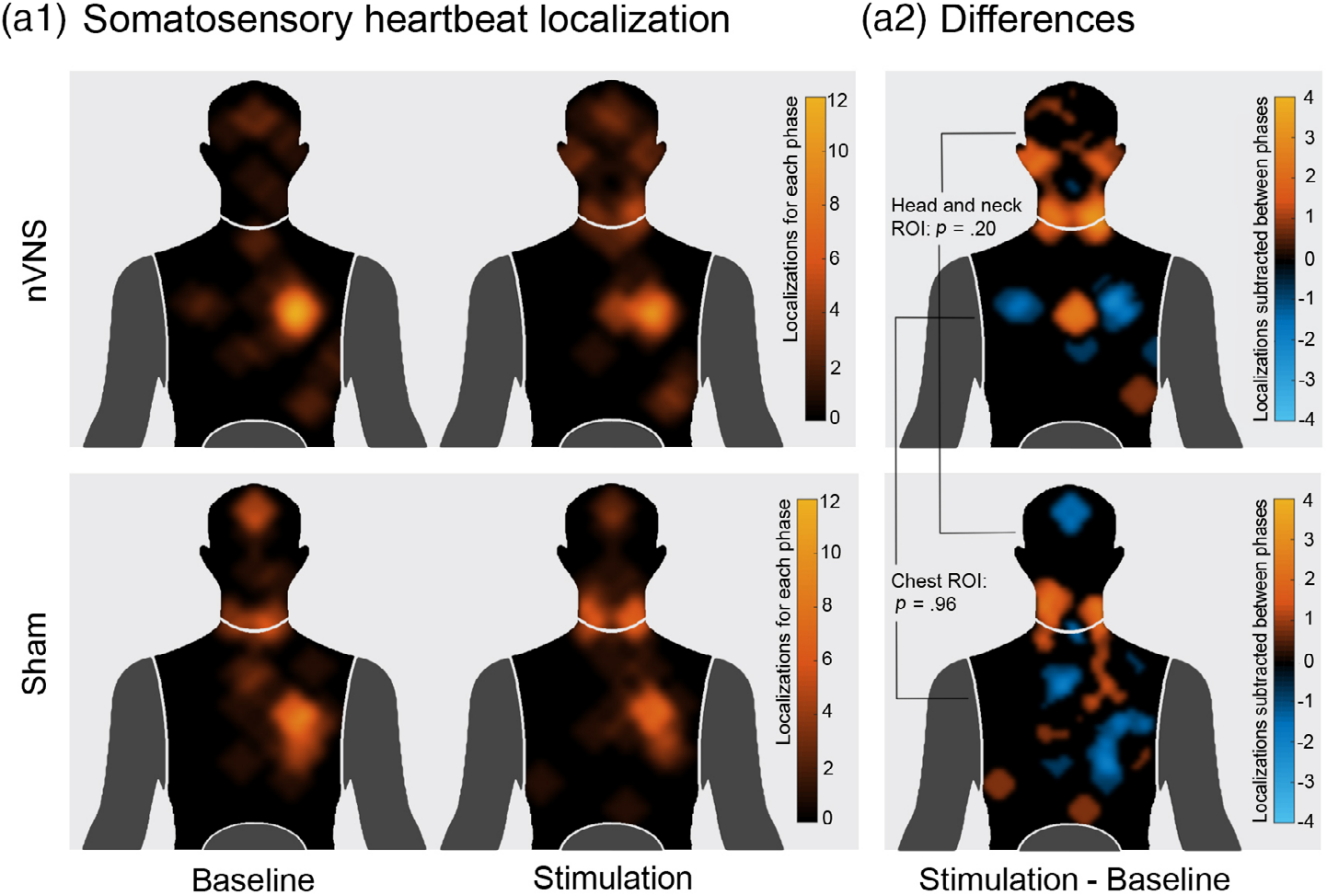
Somatosensory heartbeat perception localization. (a1) Somatosensory heartbeat localization. Locations of perceived heartbeat sensations overlapped for the corresponding group and phase. The color bar indicates the number of participants (*n*) that located their perception in a given zone, with hotter colors corresponding to a greater number of overlaps across them. Frontal view of the human body. (a2) Differences. Overlapping regions show stimulation phase minus baseline phase. The color bar indicates the differences between phases in each part of the mannequin: the yellow color indicates a greater overlap across participants during the stimulation phase, while the light blue color signals a greater overlap of participants' responses in the baseline phase. Frontal view of the human body

No significant differences between the locations of perceived heartbeat sensations were found between the nVNS (head and neck ROI: *mean* = 22.70, *SD* = 56.53; chest ROI: *mean* = −5.25, *SD* = 31.61) and the sham group (head and neck ROI: *mean* = 3.59, *SD* = 53.82; chest ROI: mean = −5.44, *SD* = 34.34) in the subtraction score between the baseline and stimulation localization (head and neck ROI: *p* = .20, *d* = .34; chest ROI: *p* = .96, *d* = .005) (see Figure 3).

### 3.5 Behavioral reaction times

Temporal distributions of the behavioral reactions did not differ significantly from uniform distribution (see Supplementary Figure 1). No significant pre–post differences in reaction times during the cardiac cycle were found.

## 4 DISCUSSION

Our study shows that direct stimulation of the vagal pathway impacts on neurocognitive markers of interoception. Relative to the sham group, the subjects undergoing nVNS exhibited selective enhancements of interoceptive performance alongside increased modulations of a cortical marker of interoception (i.e., the HEP). No between‐group differences were observed regarding the localization of the heartbeat sensations, HR or HRV (except for difference of the last but only in the second exteroceptive condition block). Thus, our findings support the critical role of the vagal pathway in interoception.

First, we found that vagal stimulation augmented interoceptive HBD accuracy. In line with conjectural claims based on computational modeling (Mourdoukoutas et al., 2018), electrophysiological studies (Nonis et al., 2017), and neuroimaging techniques (Frangos & Komisaruk, 2017), this result offers the first demonstration that the vagus nerve is directly implicated in the afferent transmission of interoceptive signals. Interestingly, the interoceptive boost on behavioral performance was observed only in the first block of the motor HBD task immediately following the stimulation, indicating a fairly rapid reduction of induced modulations. Such a fast boost‐and‐decline pattern is consistent with functional imaging evidence showing that NTS activity peaks 1 min after nVNS, quickly decreasing thereon (Frangos et al., 2015).

In this sense, previous studies have linked better performance in a motor HBD task with improved interoceptive sensitivity (Canales‐Johnson et al., 2015; de la Fuente et al., 2019; Yoris et al., 2018) as well as larger gray matter volume and greater connectivity among interoceptive hubs, including the IC (García‐Cordero et al., 2017; Salamone et al., 2018; Sedeño et al., 2014; Yoris et al., 2018). Since nVNS activates insular structures and other interoceptive areas (Frangos & Komisaruk, 2017; Mourdoukoutas et al., 2018; Nonis et al., 2017), our present results speak to a direct link between vagus nerve dynamics and activation of body‐sensing networks. Importantly, note that the effect we detected emerged while controlling for baseline variability, which rules out potential confounds related to heterogeneous interoceptive abilities across subjects.

Furthermore, this enhancement was only present in the interoceptive condition. During the exteroceptive condition, no behavioral effects were observed in either group. As in previous reports using the same task (Adolfi et al., 2017; Sedeño et al., 2014; Yoris et al., 2017; Yoris et al., 2018), the lack of exteroceptive effects suggests that the abovementioned boost may be related to neurovisceral modulations, and that it hardly constitutes an epiphenomenon driven by unspecific attentional, motivational, affective or motor effects potentially triggered by nVNS. Furthermore, the effect was present only in the *first* block of the interoceptive condition, with pre–post comparisons revealing that interoceptive sensitivity increased for the nVNS group and decreased for the sham group. The increase in interoceptive performance for both groups in the second block can be explained as a learning effect driven by task exposure, as already found in comparable studies (Melloni et al., 2013; Yoris et al., 2015). Still, further research would be needed to confirm this interpretation.

Regardless of the phase and group, participants did press with similar latency to the R‐peak (see also Galvez‐Pol et al., 2020; Kunzendorf et al., 2019; Ohl et al., 2016). This means that nVNS might not improve interoceptive processing at the precision level, despite influencing overall accuracy. From a signal‐theory perspective, this suggests that nVNS might lower the threshold for detecting a heartbeat by affecting the signal‐noise‐ratio. This lowering of the threshold is not expressed in the mean reaction time, but in the sheer number of reactions in the HEP time window. This general improvement in interoception capabilities is also reflected in the HEP curves, as a measure which is not influenced by the motor responses, but by the modulation of the subjects' attention.

That selective behavioral effect was accompanied by distinct neurophysiological signatures. HEP amplitude increased significantly in the nNVS group, whereas it actually decreased in the sham group. Note that increased HEP amplitude in the 200–500 ms window is a hallmark of improved cortical processing of afferent cardiac signals (García‐Cordero et al., 2017; Gray et al., 2007; Salamone et al., 2018; Schandry & Weitkunat, 1990). Indeed, the brain regions sensitive to cervical nVNS include the aIC and S1, both of which constitute cortical sources of the HEP (Park et al., 2018; Pollatos, Kirsch, & Schandry, 2005a). Remarkably, significant effects could only be found at the central frontal and the left frontal ROIs. This is in line with numerous findings showing that greatest modulation of HEP activity can be found on fronto‐central electrodes and that it decreases with laterality (de la Fuente et al., 2019; García‐Cordero et al., 2016; Leopold & Schandry, 2001; Marshall, Gentsch, Blum, Broering, & Schütz‐Bosbach, 2019; Salamone et al., 2018; Schandry & Montoya, 1996; Yoris et al., 2018). Considering these findings alongside our behavioral results, we propose that nVNS activates key areas of the interoceptive network and modulates putative neurophysiological markers. Accordingly, the observed HEP modulation could reflect bottom‐up facilitation of neural interoceptive processing due to nVNS. It is important to note here that the specificity of the nVNS effect is orthogonal to the observation of HEP modulations in both conditions. In contrast to the accuracy score, the HEP represents a marker of cardioceptive attention regardless of the condition.

Interestingly, short‐term vagus nerve stimulation increases firing rates of noradrenergic neurons in the locus coeruleus via the NTS (Dorr, 2006; Farrand et al., 2017). This may represent a more fine‐grained mechanism underlying the observed effect, as noradrenaline can intensify arousal states (Berridge, 2008; Foote, Bloom, & Aston‐Jones, 2017) and improve precision of sensory prediction of bottom‐up error signals (Ferreira‐Santos, 2016). Conversely, the amplitude decrease in the sham group can be attributed to top‐down or attentional effects. Recent research has shown that attentional focus shift and even pure stimulus repetition, especially during repeated presentation of external information, can diminish HEP modulations (Gentsch, Sel, Marshall, & Schütz‐Bosbach, 2019; Marshall, Gentsch, Jelinčić, & Schütz‐Bosbach, 2017; Petzschner et al., 2018). In this sense, the direct comparison between the HEP modulations of the two groups (controlled for baseline variability) revealed a significantly stronger modulation toward negativity in the nVNS group, indicating heightened interoceptive processing within 200 and 500 ms over the central‐frontal ROI and the left frontal ROI. Importantly, these regions have already been associated with HEP modulations in various populations (García‐Cordero et al., 2017; Pollatos et al., 2005a; Salamone et al., 2018; Yoris et al., 2018).

Similar HEP modulations emerged during the exteroceptive HBD condition. Though this result might seem counterintuitive at first sight, note that the HEP is continuously monitored and registered via interoceptive cortical networks even if there is no conscious heartbeat perception (Immanuel et al., 2014; Montoya et al., 1993; Schandry et al., 1986). In other words, as the HEP is triggered by the heartbeat, and this trigger is present in both conditions (interoceptive and exteroceptive), we can track interoceptive dynamics in both conditions at the electrophysiological level. In particular, given that cervical nVNS stimulates structures that are considered cortical sources of HEP (Frangos & Komisaruk, 2017; Park et al., 2018; Pollatos et al., 2005a), the bottom‐up stimulation effect in the nVNS group may also prove visible in the HEP curves of the exteroceptive condition. In fact, the HEP decrease observed in the sham group during interoception also emerged in the exteroceptive condition, further supporting the above interpretation.

Finally, both groups showed no stimulation effect in the somatosensory perception of cardiac sensations. This is consistent with studies showing that cervical nVNS primarily and directly affects vagal, but not somatosensory (somatosensory pathways comprise large‐diameter sensory fibers that project skin afferents to somatosensory regions via the dorsal columns of the spinal tract as well as small‐diameter fibers projecting via the lamina I spinothalamic pathway to the IC und ACC (Cameron, 2001; Craig, 2002a, 2002b; Olausson et al., 2002)), pathways (Frangos & Komisaruk, 2017; Mourdoukoutas et al., 2018; Nonis et al., 2017). Our results therefore suggest that cervical nVNS did not generate any differences in cardioceptive dynamics afforded by such components as baroreceptors in the carotid sinus, and that the observed effect in the nVNS group can be attributed exclusively to the vagal tract manipulation.

Taken together, our findings have a number of implications for basic and applied research on neurovisceral integration. In basic research, recent computational theories suggest that interoception lies at the core of coding processes which predict internal models of the world and of others' behavior (Barrett & Simmons, 2015; Feldman Barrett, 2017; Seth, Suzuki, & Critchley, 2012). In line with this framework, we propose that vagus‐nerve stimulation could effectively increase confidence in the reliability of incoming interoceptive signals (Shipp, Adams, & Friston, 2013), which could either help to precisely modify predictions of visceral events or to actively generate the predicted sensations (Barrett & Simmons, 2015). Here, this greater confidence in the reliability of the body's own signals would be directly expressed by higher accuracy in the HBD task. Hence, our study represents a promising meeting point toward predictive‐coding accounts of interoception processing.

Our study also carries potential clinical implications. Appropriate integration of bodily signals appears to be of central importance for adaptive cognitive and emotional processing (Critchley and Garfinkel, 2017, Critchley & Garfinkel, 2018; Farb et al., 2015; Tsakiris & Critchley, 2016). In fact, interoceptive deficits have been repeatedly observed in patients with anxiety (Paulus & Stein, 2010), affective disorders (Avery et al., 2014), anorexia nervosa (Berner et al., 2018), addictive behavior (Naqvi & Bechara, 2009), depersonalization–derealization disorder (Sedeño et al., 2014), multiple sclerosis (Salamone et al., 2018), posttraumatic stress disorder (Van Der Kolk, 2006), and somatoform disorders (Cameron, 2001).

Although specific studies are needed in these conditions, our findings suggest that nVNS could represent a promising avenue for boosting neurovisceral integration in these populations and perhaps contribute to standard clinical or pharmacological treatments. In fact, nVNS has already proven effective for addressing disorders like migraine and cluster headache (de Coo et al., 2019), epilepsy (Stefan et al., 2012) and depression (Trevizol et al., 2016). Thus, our findings open up new horizons to evaluate the potential treatment effect of nVNS on diverse conditions typified by primary or secondary interoceptive disturbances.

## 5 LIMITATIONS AND FUTURE DIRECTIONS

Our study features a number of limitations which pave the way for further research. First, we cannot make a definite statement regarding the specificity of the observed effect. This follows from the lack of an unambiguous control condition for the interoceptive condition. However, our design including a sham group suggests that the nVNS effect on interoceptive markers (behavioral and ERP) is associated to the application of the stimulation rather than to other confounding variables. Then, by including a single stimulation session before the two blocks of each condition, we were able to estimate whether nVNS effects tend to be short‐lived or long lasting. Based on our results, future studies could specifically evaluate how long the effect actually lasts and whether it can be boosted by applying more stimulations. A further limitation is that our design only tapped on cardiac monitoring, thus proving blind to other aspects of interoception. Future studies should, therefore, test the generalizability of our results to additional modes of inner‐signal processing. Note that to prevent Type‐II errors due to the small number of contrasts in our study and to favor comparability with its only direct antecedent (Villani et al., 2019), our results were not corrected for multiple comparisons. Still, this could be attempted in future replications that do warrant these controls.

It is also essential to develop experimental designs that enable checking whether somatosensory information is sufficient to maintain interoception when afferent vagal projections are interrupted. More generally, as suggested above, it would be useful to examine whether nVNS might be used in the treatment of mental health conditions characterized by interoceptive difficulties, such as depression, eating disorders, autism spectrum disorders, or alexithymia (Avery et al., 2014; Berner et al., 2018; Garfinkel et al., 2016; Wiebking & Northoff, 2015). Stimulating other structures of the afferent vagal pathway (auricular branch) with a different operating device showed longer lasting effects than those found in our study (Frangos et al., 2015). Our results therefore suggest that the effect of the one‐time gammaCore‐stimulation is short‐lived. It might be the aim of future studies to find out whether this is due to the stimulated structure of the vagus nerve or to the operating mode of the respective device. Also, to illuminate possible differences between the right and left vagal pathways, further research should examine laterality effects associated with nVNS.

## 6 CONCLUSION

This study showed that the stimulation of the cervical vagus nerve triggers modulations of key behavioral and electrophysiological markers of interoception, without affecting basic cardiodynamic variables (HR and HRV) or somatosensory sensations. Such findings support the critical role of the vagal pathway for interoception and provide possible explanatory models for the efficiency of nVNS. Future research along these lines may afford major constraints toward the development of fine‐grained models of neurovisceral integration during cognitive processing.

## CONFLICT OF INTEREST

The authors declare no conflict of interests.

## Supporting information


**Appendix** **S1:** Supplementary Information

## Data Availability

All experimental data are fully available online (Richter et al., 2020).
