# Supplementary material for: Behavioral and neurophysiological signatures of interoceptive enhancements following vagus nerve stimulation
Source: Hum Brain Mapp. 2020 Dec 16;42(5):1227–42. doi: 10.1002/hbm.25288 (PMC7927286; doi:10.1002/hbm.25288)
Supplement: Supplementary file 1 — Appendix S1: Supplementary Information [file HBM-42-1227-s001.docx]

**Behavioral and neurophysiological signatures of interoceptive enhancements following vagus nerve stimulation**

Fabian Richter, Adolfo M. García, Nicolas Rodriguez Arriagada, Adrian Yoris, Agustina Birba, David Huepe, Heinz Zimmer, Agustín Ibáñez, Lucas Sedeño

**Supplementary Material**

**1 Materials and Methods**

**1.1 Supplementary Tables**

Supplementary Table 1. Demographic, neuropsychological, mood and interoceptive sensitivity measures from the subset of participants on which the EEG analyses were calculated.

| Variables | Groups | | Statistics |
| --- | --- | --- | --- |
|  | nVNS | Sham |  |
| Demographic results | | | |
| Gender (F:M) | 17:10 | 16:12 | *X^2^* = 2.23, *p* = .14 |
| Age | 25.41 (5.28) | 25.79 (5.63) | *F* = 0.07, *p* = .80, [*η*](https://de.wikipedia.org/wiki/%CE%97-Meson)*p*^2^ = .001 |
| Education | 17.11 (2.55) | 16.46 (2.05) | *F* = 1.08, *p* = .30, [*η*](https://de.wikipedia.org/wiki/%CE%97-Meson)*p*^2^ = .020 |
| Neuropsychological assessment | | | |
| IFS global score | 25.26 (3.09) | 25.85 (2.45) | *F* = 0.44, *p* = .51, [*η*](https://de.wikipedia.org/wiki/%CE%97-Meson)*p*^2^ = .011 |
| IFS (working memory sub-index) | 6.76 (1.99) | 7.71 (1.53) | *F* = 2.74, *p* = .11, [*η*](https://de.wikipedia.org/wiki/%CE%97-Meson)*p*^2^ = .064 |
| Mood and anxiety results | | | |
| BDI-II | 7.68 (5.57) | 7.71 (5.98) | *F* = 0.00, *p* = .98, [*η*](https://de.wikipedia.org/wiki/%CE%97-Meson)*p*^2^ = .000 |
| STAI-S | 31.04 (5.24) | 30.17 (4.75) | *F* = 0.37, *p* = .55, [*η*](https://de.wikipedia.org/wiki/%CE%97-Meson)*p*^2^ = .008 |
| Interoceptive control measures | | | |
| MAIA | 87.44 (29.97) | 95.74 (18.21) | *F* = 1.15, *p* = .29, [*η*](https://de.wikipedia.org/wiki/%CE%97-Meson)*p*^2^ = .025 |
| Body mass index | 22.32 (3.31) | 22.23 (2.98) | *F* = 0.01, *p* = .92, [*η*](https://de.wikipedia.org/wiki/%CE%97-Meson)*p*^2^ = .000 |

Results are presented as mean (SD). The asterisk (*) indicates chi-square analysis, whereas the pound sign (^#^) refers to one-way ANOVAs. HR: heart rate; HRV : heart rate variability; IFS: INECO Frontal Screening battery; BDI-II: Beck Depression Inventory II; STAI-S: State Anxiety Index; MAIA: Multidimensional Assessment of Interoceptive Awareness.

Supplementary Table 2. Remaining epochs after removal of artifactual epochs.

| Condition | Phase | Block | Mean (SD) | | p-value |
| --- | --- | --- | --- | --- | --- |
|  |  |  | nVNS | Sham |  |
| Interoception | Baseline | Block 1 | 108.52 (21.65) | 104.03 (20.90) | .43 |
|  |  | Block 2 | 102.45 (20.37) | 93.21 (21.01) | .10 |
|  | Stimulation | Block 1 | 106.45 (22.48) | 97.79 (19.94) | .13 |
|  |  | Block 2 | 96.83 (20.94) | 91.34 (21.56) | .33 |
| Exteroception | Baseline | Block 1 | 106.86 (18.21) | 99.83 (14.80) | .11 |
|  |  | Block 2 | 106.52 (18.30) | 110.55 (20.47) | .43 |
|  | Stimulation | Block 1 | 104.10 (20.28) | 95.79 (20.94) | .13 |
|  |  | Block 2 | 105.31 (18.47) | 105.48 (22.35) | .97 |

Results are presented as mean (SD). The p-value means the comparison between the nVNS and the sham group.

Supplementary Table 3. Counterbalanced Scheme for the nVNS group.

| **nVNS Group** | | | | |
| --- | --- | --- | --- | --- |
| Subject # | Order of Conditions | Setup Phase | 1^st^ Stimulation | 2^nd^ Stimulation |
| 1 | Ext - int | r – l | r – l (ext) | l – r (int) |
| 2 | Ext - int | l – r | l – r (ext) | r – l (int) |
| 3 | Int- ext | r – l | r – l (int) | I – d (ext) |
| 4 | Int- ext | l – r | l – r (int) | r – l (ext) |
| 5 | Ext - int | r – l | r – l (ext) | l – r (int) |
| 6 | Ext - int | l – r | l – r (ext) | r – l (int) |
| 7 | Int- ext | r – l | r – l (int) | I – d (ext) |
| 8 | Int- ext | l – r | l – r (int) | r – l (ext) |
| 9 | Ext - int | r – l | r – l (ext) | l – r (int) |
| 10 | Ext - int | l – r | l – r (ext) | r – l (int) |
| 11 | Int- ext | r – l | r – l (int) | I – d (ext) |
| 12 | Int- ext | l – r | l – r (int) | r – l (ext) |
| 13 | Ext - int | r – l | r – l (ext) | l – r (int) |
| 14 | Ext - int | l – r | l – r (ext) | r – l (int) |
| 15 | Int- ext | r – l | r – l (int) | I – d (ext) |
| 16 | Int- ext | l – r | l – r (int) | r – l (ext) |
| 17 | Ext - int | r – l | r – l (ext) | l – r (int) |
| 18 | Ext - int | l – r | l – r (ext) | r – l (int) |
| 19 | Int- ext | r – l | r – l (int) | I – d (ext) |
| 20 | Int- ext | l – r | l – r (int) | r – l (ext) |
| 21 | Ext - int | r – l | r – l (ext) | l – r (int) |
| 22 | Ext - int | l – r | l – r (ext) | r – l (int) |
| 23 | Int- ext | r – l | r – l (int) | I – d (ext) |
| 24 | Int- ext | l – r | l – r (int) | r – l (ext) |
| 25 | Ext - int | r – l | r – l (ext) | l – r (int) |
| 26 | Ext - int | l – r | l – r (ext) | r – l (int) |
| 27 | Int- ext | r – l | r – l (int) | I – d (ext) |
| 28 | Int- ext | l – r | l – r (int) | r – l (ext) |
| 29 | Ext - int | r – l | r – l (ext) | l – r (int) |
| 30 | Ext - int | l – r | l – r (ext) | r – l (int) |
| 31 | Int- ext | r – l | r – l (int) | I – d (ext) |
| 32 | Int- ext | l – r | l – r (int) | r – l (ext) |
| 33 | Ext - int | l – r | l – r (ext) | r – l (int) |
| 34 | Int- ext | l – r | l – r (int) | r – l (ext) |
| 35 | Ext - int | l – r | l – r (ext) | r – l (int) |

Ext = Exteroceptive Condition, Int = Interoceptive Condition, l = left, r = right.

Supplementary Table 4. Counterbalanced Scheme for the Sham group.

| **Sham stimulation group** | | | | |
| --- | --- | --- | --- | --- |
| Subject # | Order of Conditions | Setup Phase | 1^st^ Stimulation | 2^nd^ Stimulation |
| 1 | Ext - int | r - l | r – l (ext) | l – r (int) |
| 2 | Ext - int | l – r | l – r (ext) | r – l (int) |
| 3 | Int- ext | r - l | r – l (int) | I – d (ext) |
| 4 | Int- ext | l - r | l – r (int) | r – l (ext) |
| 5 | Ext - int | r - l | r – l (ext) | l – r (int) |
| 6 | Int- ext | r - l | r – l (int) | I – d (ext) |
| 7 | Ext - int | r - l | r – l (ext) | l – r (int) |
| 8 | Ext - int | l - r | l – r (ext) | r – l (int) |
| 9 | Int- ext | r - l | r – l (int) | I – d (ext) |
| 10 | Int- ext | l - r | l – r (int) | r – l (ext) |
| 11 | Ext - int | r - l | r – l (ext) | l – r (int) |
| 12 | Ext - int | l - r | l – r (ext) | r – l (int) |
| 13 | Int- ext | r - l | r – l (int) | I – d (ext) |
| 14 | Int- ext | l - r | l – r (int) | r – l (ext) |
| 15 | Ext - int | r - l | r – l (ext) | l – r (int) |
| 16 | Ext - int | l - r | l – r (ext) | r – l (int) |
| 17 | Int- ext | l - r | l – r (int) | r – l (ext) |
| 18 | Ext - int | r - l | r – l (ext) | l – r (int) |
| 19 | Ext - int | l - r | l – r (ext) | r – l (int) |
| 20 | Int- ext | r - l | r – l (int) | I – d (ext) |
| 21 | Ext - int | r - l | r – l (ext) | l – r (int) |
| 22 | Ext - int | l - r | l – r (ext) | r – l (int) |
| 23 | Int- ext | r - l | r – l (int) | I – d (ext) |
| 24 | Int- ext | l - r | l – r (int) | r – l (ext) |
| 25 | Ext - int | r - l | r – l (ext) | l – r (int) |
| 26 | Ext - int | l - r | l – r (ext) | r – l (int) |
| 27 | Int- ext | r - l | r – l (int) | I – d (ext) |
| 28 | Int- ext | l - r | l – r (int) | r – l (ext) |

Ext = Exteroceptive Condition, Int = Interoceptive Condition, l = left, r = right.

Supplementary Table 5. Averaged HEP values for the significant time windows of nVNS vs. sham comparisons

|  |  | Sham | nVNS | ms |
| --- | --- | --- | --- | --- |
| Interoception | ROI central | 0.27 (0.14) | -0.42 (0.12) | 409-463 |
|  | ROI left | 0.40 (0.15) | -0.37 (0.13) | 397-467 |
| Exteroception | ROI central | 0.43 (0.12) | -0.61 (0.06) | 370-393 |
|  | ROI left | 0.49 (0.12) | -0.42 (0.06) | 366-428 |
|  | ROI left | 0.45 (0.06) | -0.31 (0.02) | 456-499 |

Numbers in brackets indicate Standard deviations, ms = milliseconds.

Supplementary Table 6. Mean pre-post differences (Accuracy) and results of paired pre-post permutation analyses (5000 permutations, p < .05)

|  | | Block 1 | Block 2 |
| --- | --- | --- | --- |
| Mean of pre-post difference (Accuracy) | nVNS group | + 0.06 (p = .01) | + 0.04 (p = .054) |
|  | Sham group | - 0.02 (p = .11) | + 0.04 (p = .02) |

Supplementary Table 7. Mean baseline differences (Accuracy) between groups (permutation analyses; 5000 permutations, p < .05)

|  | | Block 1 | Block 2 |
| --- | --- | --- | --- |
| Mean baseline differences between nVNS and Sham (Accuracy) | Interoceptive condition | 0.08 (p = .09) | 0.05 (p = .35) |
|  | Exteroceptive condition | 0.03 (p = .66) | 0.01 (p = .84) |

Supplementary Table 8. Spearman correlations between HEP and HR data

| Spearman’s Rho  HR x HEP | | Interoceptive | Exteroceptive |
| --- | --- | --- | --- |
| nVNS | Baseline | .10 (p = .63) | -.09 (p = .64) |
|  | Stimulation | .18 (p = .38) | .31 (p = 11) |
| Sham | Baseline | .01 (p = .96) | .06 (p = .77) |
|  | Stimulation | -.09 (p = .64) | .05 (p = .81) |

Supplementary Table 9. Spearman correlations between HEP and HRV data

| **Spearman’s Rho**  **HRV x HEP** | | Interoceptive | Exteroceptive |
| --- | --- | --- | --- |
| nVNS | Baseline | -.10 (p = .63) | .31 (p = .12) |
|  | Stimulation | .25 (p = .21) | .004 (p = .98) |
| Sham | Baseline | -.22 (p = .28) | -.11 (p = .58) |
|  | Stimulation | .21 (p = .30) | -.20 (p = .31) |

Supplementary Table 10. Spearman’s correlations between electrophysiological and behavioral data

| **Spearman’s Rho: Mean HEP value of time window x mean Accuracy** | Rho (p-value) | Significant time window (at Biosemi B22 electrode) |
| --- | --- | --- |
| Exp_bl | -.06 (.77) | 303 – 362 ms |
| Exp_est | .11 (.58) |  |
| Sham_bl | -.12 (.53) | 405 – 436 ms |
| Sham_est | .06 (.78) |  |

Supplementary Figure 1


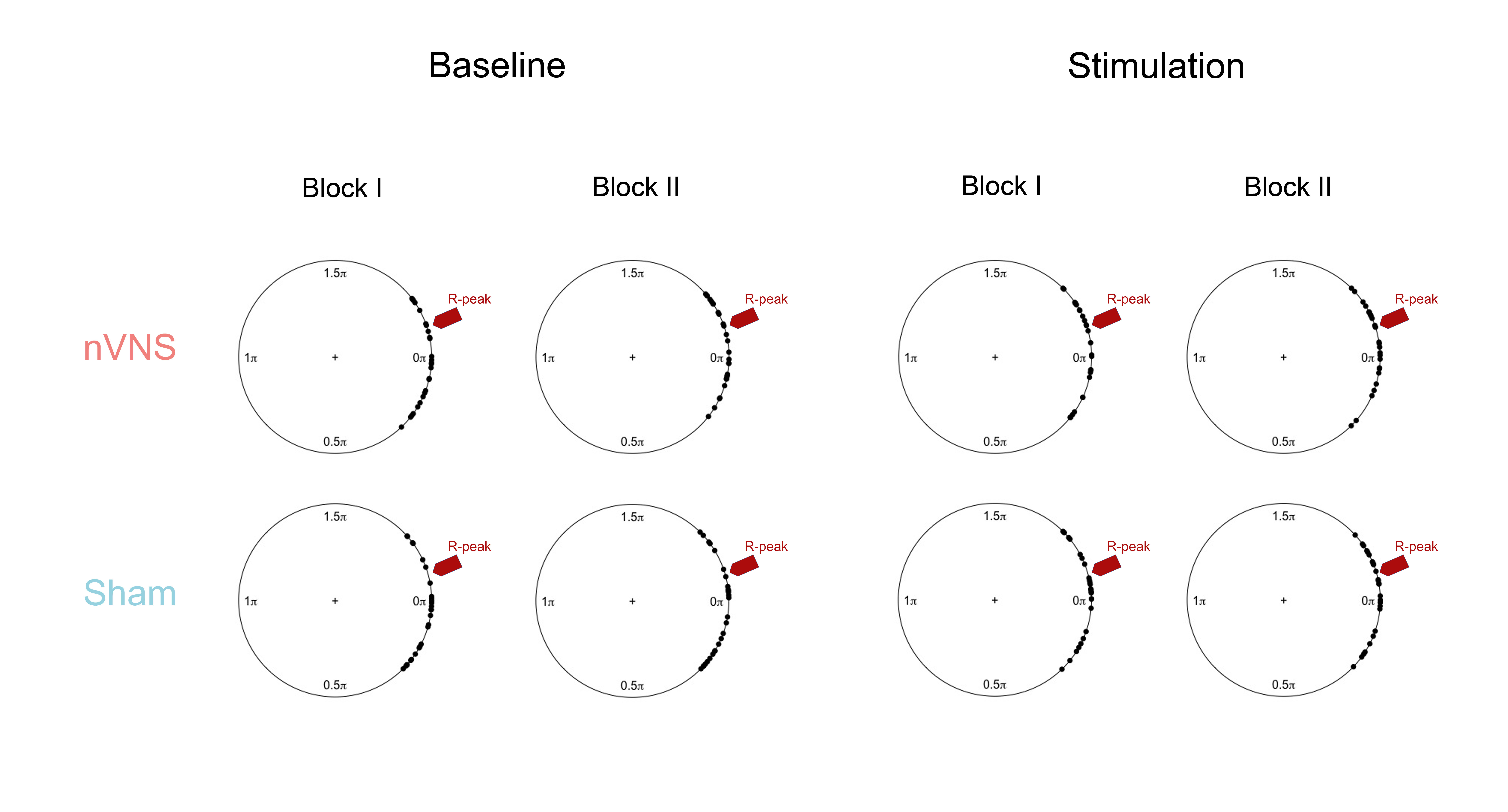


**Supplementary Figure 1: Circular Analyses**. Mean reaction times are plotted for each subject. The cardiac phase cycles clockwise round the circle, with R-peaks occurring at 72°. Rayleigh tests (Landler et al., 2018) showed that the temporal distributions of behavioral reactions did not differ significantly from uniform distribution. Watson tests (Mardia & Jupp, 2008) revealed no significant pre-post differences in reaction times.

**1.2 Heartbeat Detection Task**

The equation used to assess performance on the HBD Task was:

1-((Recorded Heartbeats – Total of Correct Answers)/ Recorded Heartbeats))

This accuracy index normalizes the subjects correct responses based on the total amount of heartbeats. It also allows us to compare participants’ performance without the bias of heart rate differences. The original method proposed by Schandry (Schandry et al., 1986) employs the total amount of mental heartbeats counted and the total number of heartbeats recorded as measures for his index. This method can be referred to as mental tracking paradigm. We modified this method by applying a motor tracking paradigm. We have done this in order to take into account a widespread criticism of the mental tracking task (Desmedt et al., 2018; Ring et al., 2015; Ring & Brener, 1996, 2018; Zamariola et al., 2018) and by doing so, to increase the validity of the HBDT. This tracking method (Couto et al., 2015; García-Cordero et al., 2016, 2017; Melloni et al., 2013; Sedeño et al., 2014; Yoris et al., 2015) allows us to classify the motor responses (tapping on the keyboard) of the participants into correct and incorrect answers. To do so, every motor response was compared with a specific time window subsequently to every recorded heartbeat, whereby the length of the time window is determined by the subjects’ heart rate: 0.750 milliseconds after the R wave, for a heart frequency less than 69.76; 0.6 after, for frequencies between 69.75 and 94.25; 0.4 milliseconds after, for frequencies higher than 94.25. If the tapping input was temporarily located within the corresponding time window for a given beat, the response was considered as correct. An accuracy Index was calculated for every condition, varying from 0 to 1, with high scores indicating only small differences between correct answers and recorded heartbeats (Canales-Johnson et al., 2015; Couto et al., 2015; García-Cordero et al., 2016, 2017; Melloni et al., 2013; Sedeño et al., 2014; Yoris et al., 2015).

For the exteroceptive condition, the same equation and procedure was applied. The difference, however, was that the time window was identical for each heartbeat-sound played. It started 100 milliseconds before the tone and ended 600 milliseconds after the tone.

**2 References**

Canales-Johnson, A., Silva, C., Huepe, D., Rivera-Rei, Á., Noreika, V., Del Carmen Garcia, M., Silva, W., Ciraolo, C., Vaucheret, E., Sedeño, L., Couto, B., Kargieman, L., Baglivo, F., Sigman, M., Chennu, S., Ibáñez, A., Rodríguez, E., & Bekinschtein, T. A. (2015). Auditory feedback differentially modulates behavioral and neural markers of objective and subjective performance when tapping to your heartbeat. *Cerebral Cortex*, *25*, 4490–4503. https://doi.org/10.1093/cercor/bhv076

Couto, B., Adolfi, F., Sedeño, L., Salles, A., Canales-Johnson, A., Alvarez-Abut, P., Garcia-Cordero, I., Pietto, M., Bekinschtein, T., Sigman, M., Manes, F., & Ibanez, A. (2015). Disentangling interoception: Insights from focal strokes affecting the perception of external and internal milieus. *Frontiers in Psychology*, *6*, 503. https://doi.org/10.3389/fpsyg.2015.00503

Desmedt, O., Luminet, O., & Corneille, O. (2018). The heartbeat counting task largely involves non-interoceptive processes: Evidence from both the original and an adapted counting task. *Biological Psychology*. https://doi.org/10.1016/j.biopsycho.2018.09.004

García-Cordero, I., Esteves, S., Mikulan, E. P., Hesse, E., Baglivo, F. H., Silva, W., García, M. del C., Vaucheret, E., Ciraolo, C., García, H. S., Adolfi, F., Pietto, M., Herrera, E., Legaz, A., Manes, F., García, A. M., Sigman, M., Bekinschtein, T. A., Ibáñez, A., & Sedeño, L. (2017). Attention, in and out: Scalp-level and intracranial EEG correlates of interoception and exteroception. *Frontiers in Neuroscience*, *11*, 411. https://doi.org/10.3389/fnins.2017.00411

García-Cordero, I., Sedeño, L., de la Fuente, L., Slachevsky, A., Forno, G., Klein, F., Lillo, P., Ferrari, J., Rodriguez, C., Bustin, J., Torralva, T., Baez, S., Yoris, A., Esteves, S., Melloni, M., Salamone, P., Huepe, D., Manes, F., García, A. M., & Ibañez, A. (2016). Feeling, learning from and being aware of inner states: interoceptive dimensions in neurodegeneration and stroke. *Philosophical Transactions of the Royal Society B: Biological Sciences*, *371*(1708), 20160006. https://doi.org/10.1098/rstb.2016.0006

Landler, L., Ruxton, G. D., & Malkemper, E. P. (2018). Circular data in biology: advice for effectively implementing statistical procedures. *Behavioral Ecology and Sociobiology*. https://doi.org/10.1007/s00265-018-2538-y

Mardia, K. V., & Jupp, P. E. (2008). Directional Statistics. In *Directional Statistics*. https://doi.org/10.1002/9780470316979

Melloni, M., Sedeño, L., Couto, B., Reynoso, M., Gelormini, C., Favaloro, R., Canales-Johnson, A., Sigman, M., Manes, F., & Ibanez, A. (2013). Preliminary evidence about the effects of meditation on interoceptive sensitivity and social cognition. *Behavioral and Brain Functions*, *9*, 47. https://doi.org/10.1186/1744-9081-9-47

Ring, C., & Brener, J. (1996). Influence of beliefs about heart rate and actual heart rate on heartbeat counting. *Psychophysiology*, *33*(5), 541–546. https://doi.org/10.1111/j.1469-8986.1996.tb02430.x

Ring, C., & Brener, J. (2018). Heartbeat counting is unrelated to heartbeat detection: A comparison of methods to quantify interoception. *Psychophysiology*, *55*, e13084. https://doi.org/10.1111/psyp.13084

Ring, C., Brener, J., Knapp, K., & Mailloux, J. (2015). Effects of heartbeat feedback on beliefs about heart rate and heartbeat counting: A cautionary tale about interoceptive awareness. *Biological Psychology*, *104*, 193–198. https://doi.org/10.1016/j.biopsycho.2014.12.010

Schandry, R., Sparrer, B., & Weitkunat, R. (1986). From the heart to the brain: A study of heartbeat contingent scalp potentials. *International Journal of Neuroscience*, *30*, 261–275. https://doi.org/10.3109/00207458608985677

Sedeño, L., Couto, B., Melloni, M., Canales-Johnson, A., Yoris, A., Baez, S., Esteves, S., Velásquez, M., Barttfeld, P., Sigman, M., Kichic, R., Chialvo, D., Manes, F., Bekinschtein, T. A., & Ibanez, A. (2014). How do you feel when you can’t feel your body? Interoception, functional connectivity and emotional processing in depersonalization-derealization disorder. *PLoS ONE*, *9*, e98769. https://doi.org/10.1371/journal.pone.0098769

Yoris, A., Esteves, S., Couto, B., Melloni, M., Kichic, R., Cetkovich, M., Favaloro, R., Moser, J., Manes, F., Ibanez, A., & Sedeño, L. (2015). The roles of interoceptive sensitivity and metacognitive interoception in panic. *Behavioral and Brain Functions*, *8*, 11–14. https://doi.org/10.1186/s12993-015-0058-8

Zamariola, G., Maurage, P., Luminet, O., & Corneille, O. (2018). Interoceptive accuracy scores from the heartbeat counting task are problematic: Evidence from simple bivariate correlations. *Biological Psychology*, *137*, 12–17. https://doi.org/10.1016/j.biopsycho.2018.06.006
